# What most captures the physician's interest when evaluating a multiparameter monitor in a Neonatal ICU? – A simulation study

**DOI:** 10.1016/j.jped.2026.101548

**Published:** 2026-04-26

**Authors:** Simone Yumi Tsuji Assunção, Marina Carvalho de Moraes Barros, Roberto Gonçalves de Magalhães Júnior, Pâmella Cristina do Prado Franco, Beatriz Mesquita Mello, Mandira Daripa Kawakami, Ana Silvia Scavacini Marinonio, Adriana Sanudo, Milton Harumi Miyoshi, Maria Fernanda de Almeida, Tatiany Marcondes Heiderich, Rafael Nobre Orsi, Carlos Eduardo Thomaz, Ruth Guinsburg

**Affiliations:** a Universidade Federal de São Paulo, Escola Paulista de Medicina, Department of Pediatrics, Division of Neonatal Medicine, São Paulo, SP, Brazil; b Centro Universitario FEI, Department of Electrical Engineering, Image Processing Laboratory, São Bernardo do Campo, SP, Brazil; c Universidade Federal de São Paulo, Escola Paulista de Medicina, Department of Preventive Medicine, São Paulo, SP, Brazil

**Keywords:** Clinical decision making, Eye tracking technology, Neonatal intensive care units, Newborn, Vital signs

## Abstract

**Objective:**

To assess the gaze of physicians in the Neonatal ICU setting when evaluating a multiparameter monitor to determine the presence/absence of vital sign alterations.

**Methods:**

Pediatricians’ gaze was tracked when they evaluated a 30-second video of a multiparametric monitor on a screen. They assessed one of three scenarios: Normal, Tachycardia, or Hyperoxia. Eight areas of interest (AOI) were defined: Heart rate(HR), Electrocardiogram(ECG), SpO₂, Pulse wave(PulseW), Respiratory rate(RR), Respiration curve(Resp.C), Blood pressure(BP), and Temperature(Temp.). Gaze fixation on each AOI was analyzed in three periods:0–3, 0–5, 0–10 s. Generalized estimating equations were used to evaluate the effect of the parameters/scenarios on the participants' likelihood of gaze fixation on each AOI.

**Results:**

Eighty physicians (86% female; 35.3 ± 8.2 years old) were evaluated. At 0–3 s, 0–5 s, and 0–10 s, the percentage of physicians who fixed their gaze on each AOI was, respectively:HR: 93, 96, 98%; ECG: 73, 79, 89%; SpO2: 75, 94, 95%; PulseW: 86, 88, 98%; RR: 48, 80, 95%; Resp.C: 26, 49, 73%; BP: 35, 63, 98%; Temp: 31, 53, 91%. Compared with HR, the likelihood of gaze fixation was lower at 0–3 s, for ECG (*Odds Ratio*–OR: 0.21), SpO2 (OR: 0.24), RR (OR: 0.07), RespC (OR: 0.03), BP (OR: 0.04) and Temp (OR: 0.04); at 0–5 s, for ECG (OR: 0.14), RR (OR; 0.16), Resp.C (OR: 0.04), BP (OR: 0.07) and Temp (OR: 0.04); and at 0–10 s, only for Resp.C (OR: 0.07).

**Conclusion:**

In the neonatal ICU setting, when assessing the monitor, HR attracts physicians’ initial attention, followed by oximetry parameters.

## Introduction

Approximately one in ten newborns requires intensive care [1]. Monitoring vital signs is crucial for the effective management of these patients. Analog monitors, which were initially used, have now largely been replaced by digital systems that incorporate a wider range of parameters [2]. These digital monitors allow continuous and simultaneous tracking of vital signs, including heart rate, respiratory rate, oxygen saturation, blood pressure, and temperature, as well as the assessment of pulse waveforms, electrocardiograms, and respiratory movements. By integrating this information with the patient’s clinical history, physical examination, and laboratory results, physicians can provide care that is both more effective and safer [3].

Understanding physicians’ visual attention when evaluating multiparameter monitors may enhance training and optimize clinical decision-making. Eye-tracking technology has emerged as a valuable tool for analyzing visual behavior, providing insights into how clinicians interact with complex visual information. Its application has contributed to the assessment and improvement of training in clinical practice by identifying attention patterns and highlighting areas that require increased focus [4]. However, few studies have specifically investigated how healthcare professionals allocate visual attention when interpreting data from multiparameter monitors.

Therefore, the objective of this study was to assess the visual attention of pediatricians when evaluating the display of a multiparameter monitor to determine the presence or absence of alterations in vital signs or curves.

## Methods

### Population, study environment and eye tracking system

The authors included physicians who had previous experience with neonatal intensive care, including pediatricians, neonatologists, pediatric intensivists, second- and third-year pediatric residents and neonatal or pediatric intensive care fellows. Physicians whose eye signal capture time was <70% of the experiment time were excluded [5]. Participants were also asked if they had experience with the monitor used in the study (Efficia CM 120®; GamaCamp, Campinas, São Paulo, Brazil).

The study was conducted in a closed room with controlled artificial lighting. Participants were comfortably seated in a chair in front of a computer screen. The distance and height between the vision tracking device reader - Tobii TX300 (Tobii Technology AB, Danderyd, Sweden) and the participant were adjusted.

### Study design

At the beginning of the experiment, after giving instructions to the participants and calibrating the Tobii TX300, one of three different scenarios was randomly selected to be presented: Monitor 1 (M1) - normal vital signs; Monitor 2 (M2) – tachycardia; and Monitor 3 (M3) – hyperoxia. Table 1 shows the vital signs displayed on the monitor panel in each scenario. The authors selected three common clinical conditions encountered in Neonatal Intensive Care Units: normal vital signals, tachycardia and hyperoxia. While hypoxia is widely recognized as a life-threatening event that prompts an immediate clinical response, hyperoxia typically presents less acutely, despite its well-established association with adverse long-term outcomes such as bronchopulmonary dysplasia and retinopathy of prematurity. The authors therefore explored whether hyperoxia would also capture participants’ visual attention, even in the absence of an immediate life-threatening presentation.

**Table 1:** Vital signs displayed on the multiparameter monitor panel in each scenario.

	M1: Normal	M2: Tachycardia	M3: Hyperoxia
Heart rate (bpm)	130	185	137
Oxygen saturation (%)	94	92	97
Respiratory rate (mpm)	50	50	50
Blood pressure [systolic/diastolic (mean)] (mmHg)	50/31 (38)	50/31 (38)	50/31 (38)
Temperature ( °C)	37.0	37.0	37.0

M, Monitor; bpm,beats per minute; mpm, movements per minute; mmHg, millimeters of mercury; C, degrees Celsius.

A clinical case was developed for each scenario. The three cases differed only in gestational age and birth weight. "You are on-call in a Neonatal Intensive Care Unit and you are assessing a preterm infant 10 h old at 33 weeks’ gestation and birth weight of 1800 g. The patient is receiving mechanical ventilation by tracheal tube, and you are assessing the vital signs on a multiparameter monitor". For the second and third clinical cases, the gestational age was 31 and 34 weeks, and the birth weight was 1600 and 2000 g, respectively.

The experiment consisted of a sequence of three screens. On the first screen, an image from the multiparameter monitor was displayed for 20 s, allowing participants to familiarize themselves with the image. On the second screen, the clinical case was presented, with free time for reading. On the third screen, a video from the multiparameter monitor was shown for 30 s, and during this period, the participant's gaze was tracked using the Tobii TX300 device. The vision tracking equipment consists of a 23-inch thin-film transistor monitor with an infrared illumination system and two capture cameras located at the bottom of the equipment. The equipment can capture data at a frequency of 300 Hz, with a minimum fixation duration of 60 milliseconds and a maximum scatter threshold of 0.5 degrees [6].

### Recorded data

**Figure 1:**
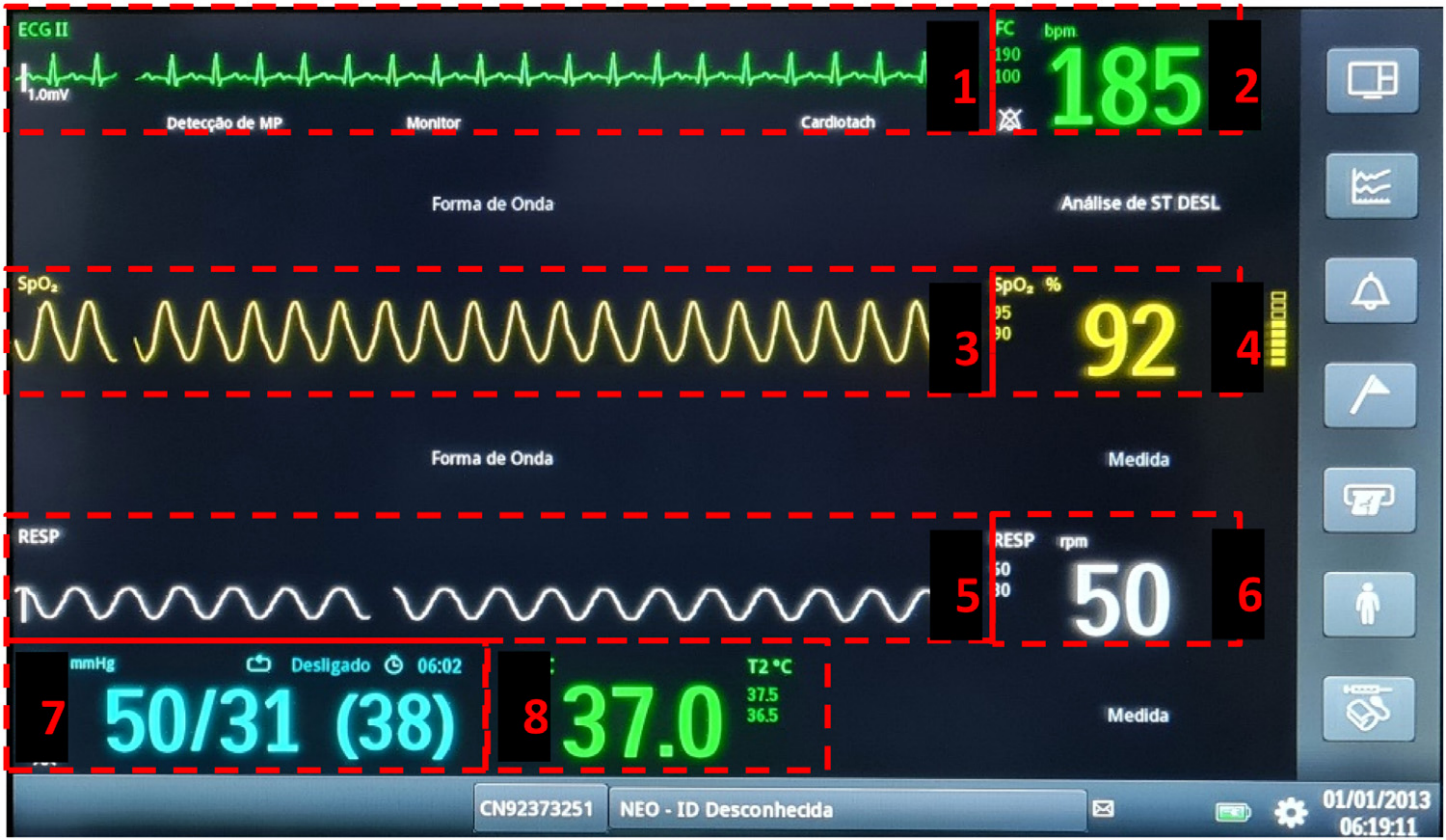
Areas of interest on the multiparameter monitor panel, for evaluating participants' visual tracking: 1: Electrocardiogram; 2: Heart rate; 3: Pulse wave; 4: Oxygen saturation; 5: Respiration Curve; 6: Respiratory rate; 7: Blood pressure (systolic, diastolic, mean); 8: Temperature.

To assess the vision tracking outcome, eight areas of interest were delimited on the monitor panel: Heart Rate (HR), Electrocardiogram (ECG), Oxygen Saturation (SpO_2_), Pulse Wave (PulseW), Respiratory Rate (RR), Respiration Curve (Resp.C), Blood Pressure (BP), and Temperature (Temp.) (Figure 1).

The participant’s gaze was tracked throughout the entire experiment. For analytical purposes, three time windows were defined: 0–3, 0–5, and 0–10 s. Within the 10-second observation window, the segmentation of the analyses into the 0–3, 0–5, and 0–10 s intervals was exploratory, given the lack of prior data on this specific issue. These intervals were not sequential; rather, they were cumulative, with each one encompassing the previous. The first window (0–3 s) captured the participant’s initial orienting response. The second window (0–5 s) included the initial interest as well as the subsequent gaze shifts. The third window (0–10 s) encompassed the two earlier intervals and allowed for a broader assessment of how visual attention explored the experimental display over time. Visual fixation was defined as the time the eyes remain relatively still, focusing on a specific point, long enough for the visual system (pupil, lens, retina, and optic nerve) to receive information about what is being looked at, at least 60 miliseconds [7].

### Sample size and data analysis

A convenience sample was studied. The sample size calculation was based on a previous study that evaluated physicians' visual tracking when observing the faces of newborns at rest, in which, on average, 75% of participants fixed their gaze on the upper and lower regions of the face [8]. Considering an alpha risk of 5% and a margin of error of 10%, 73 participants were estimated. Assuming a loss rate of 15% due to possible failure in capturing the ocular signal, the final sample size was adjusted to 86 participants.

Numerical variables were expressed as mean and standard deviation or median and interquartile range, depending on data distribution. Categorical variables were expressed as the number of events and the percentage. For each of the three predefined time windows, data were analyzed using Generalized Estimating Equations (GEE), an approach appropriate for repeated-measures designs. This method accounts for the correlation between observations from the same participant, as physicians’ visual attention was measured repeatedly over time. The model examined the effects of Parameter (the different vital signs displayed on the monitor), Scenario (M1, M2, and M3), and the interaction between these factors on the likelihood of gaze fixation within each area of interest.

The chance of doctors fixating on each parameter was compared with the chance of fixating on heart rate. The heart rate was chosen as the parameter for comparison because it was the one that initially attracted the physicians' attention when evaluating the multiparameter monitor panel. The results are expressed as odds ratios (OR) and 95% confidence intervals (95% CI).

In an additional analysis, physician experience (categorized into doctors with or without a complete fellowship in neonatology or pediatric intensive care) was included as a covariate. Interaction terms between experience and both parameters and scenario (M1, M2 and M3) were tested using the same GEE framework to assess potential effect modification.

Statistical analysis was performed using STATA/MP 18.0 software (StataCorp, 2023. College Station, TX, USA: StataCorp LLC).

This study was conducted at Escola Paulista de Medicina - Federal University of São Paulo, from February to August 2022. The study was approved by the Ethics Review Boards Research Ethics Committee of Federal University of São Paulo under CAAE No 52925621.1.0000.5505

Participants gave informed consent to participate in the study before taking part.

The study was performed in accordance with the Declaration of Helsinki.

## Results

The study enrolled 88 physicians. Of these, 8 (9.1%) were excluded due to ocular signal capture below 70% at one or more studied periods. Therefore, 80 physicians were included and their main characteristics are described in Table 2. Among these physicians, 25 (31%) evaluated M1, 27 (34%) M2, and 28 (35%) evaluated M3. The ocular capture rate at the analyzed periods was: 98 ± 5% in the 0–3 s period, 97 ± 4% in the 0–5 s period, and 96 ± 5% in the 0–10 s period.

**Table 2:** Characteristics of the 80 studied physicians.

	Physicians studied
Age (years)	35 ± 8
Female	69 (86%)
High economic level^1^	71 (89%)
Married	29 (36%)
Years of graduation	6 (4 - 13)
Pediatric Residency	71 (89%)
Years since completion of Pediatric Residency^2^	4 (1 - 11)
Residency in Neonatology/Pediatric ICU	45 (56%)
Years since Residency in Neonatology/Pediatric ICU^3^	5 (2 - 16)
Years of experience - Neonatal or Pediatric ICU	4 (2 - 11)
Weekly workload in ICU (hours)	41(24 - 60)
Experience with Efficia CM120® multiparameter monitor^4^	67 (84%)

Categorical data are expressed in number (%) and numerical variables in mean ± standard deviation or median and interquartile range. ICU, Intensive Care Unit.

1 ABEP, 2020.

2 data from 71 physicians.

3 data from 45 physicians.

4 GamaCamp, Campinas, São Paulo, Brazil.

Table 3 presents the number of physicians who fixed their gaze on each parameter of the monitor in the three studied periods (0–3 s, 0–5 s, and 0–10 s) and in the three scenarios presented. Multiple comparisons of the likelihood of fixation in each parameter, compared to heart rate, are presented in Table 4.

**Table 3:** Number (%) of physicians who fixed their gaze on the different parameters of the monitor, in the three times studied and in the three clinical cases presented.

	0–3 s	0–5 s	0–10 s
Normal vital signs			
Heart rate	23 (92%)	25 (100%)	25 (100%)
Electrocardiogram	13 (52%)	16 (64%)	21 (84%)
Oxygen saturation	22 (88%)	23 (92%)	24 (96%)
Pulse wave	22 (88%)	22 (88%)	23 (92%)
Respiratory rate	12 (48%)	22 (88%)	24 (96%)
Respiration curve	6 (24%)	14 (56%)	18 (72%)
Blood pressure	10 (40%)	16 (64%)	25 (100%)
Temperature	7 (28%)	12 (48%)	24 (96%)
Tachycardia			
Heart rate	26 (96%)	26 (96%)	26 (96%)
Electrocardiogram	21 (78%)	23 (85%)	25 (93%)
Oxygen saturation	21 (78%)	27 (100%)	27 (100%)
Pulse wave	22 (82%)	23 (85%)	27 (100%)
Respiratory rate	17 (63%)	24 (89%)	27 (100%)
Respiration curve	8 (30%)	11 (41%)	20 (74%)
Blood pressure	9 (33%)	18 (67%)	27 (100%)
Temperature	11 (41%)	16 (59%)	27 (100%)
Hyperoxia			
Heart rate	25 (89%)	26 (93%)	27 (96%)
Electrocardiogram	24 (86%)	24 (86%)	25 (89%)
Oxygen saturation	17 (61%)	25 (89%)	25 (89%)
Pulse wave	25 (89%)	25 (89%)	28 (100%)
Respiratory rate	9 (32%)	18 (64%)	25 (89%)
Respiration curve	7 (25%)	14 (50%)	20 (71%)
Blood pressure	9 (32%)	16 (57%)	26 (93%)
Temperature	7 (25%)	14 (50%)	22 (79%)

**Table 4:** Multiple comparisons between the chances of physicians fixing their gaze at each time studied, between each parameter and heart rate, adjusted for monitor and other parameters.

Parameter	0–3s	0–5s	0–10s
ECG vs. HR	0.21 (0.09 - 0.51) p < 0.001	0.14 (0.04 - 0.50) p = 0.002	0.20 (0.04 - 1.01) p = 0.052
SpO_2_ vs. HR	0.24 (0.10 - 0.60) p = 0.002	0.58 (0.16 - 2.16) p = 0.421	0.49 (0.12 - 2.06) p = 0.328
PulseW vs. HR	0.51 (0.17 - 1.54) p = 0.233	0.27 (0.07 - 1.08) p = 0.065	1.00 (0.13 - 7.56) p = 1.000
RR vs. HR	0.07 (0.03 - 0.19) p < 0.001	0.16 (0.04 - 0.59) p = 0.006	0.49 (0.08 - 2.85) p = 0.425
RespC vs. HR	0.03 (0.01 - 0.08) p < 0.001	0.04 (0.01 - 0.12) p < 0.001	0.07 (0.02 - 0.29) p < 0.001
BP vs. HR	0.04 (0.02 - 0.12) p < 0.001	0.07 (0.02 - 0.24) p < 0.001	1.00 (0.13 - 7.56) p = 1.000
Temp. vs. HR	0.04 (0.01 - 0.10) p < 0.001	0.04 (0.01 - 0.15) p < 0.001	0.27 (0.05 - 1.39) p = 0.117

Results expressed as Odds Ratio (95% confidence interval).

HR, heart rate; ECG, electrocardiogram; SpO_2_, oxygen saturation; PulseW, pulse wave; RR, respiratory rate; Resp.C, Respiration curve; BP, blood pressure; Temp., temperature.

At 0–3 s, there was no effect on Scenario alone (p = 0.358) nor an interaction between Parameter and Scenario (p = 0.088). However, Parameters had a significant effect (p < 0.001) on the likelihood of physicians fixating on each area of interest. Compared with heart rate, the likelihood of gaze fixation was lower for electrocardiogram (OR = 0.21; 95% CI: 0.09–0.51), oxygen saturation (0.24; 0.10–0.60), respiratory rate (0.07; 0.02–0.19), respiratory curve (0.03; 0.01–0.08), blood pressure (0.04; 0.02–0.12), and temperature (0.04; 0.01–0.10).

At 0–5 s, the interaction between Parameter and Scenario could not be tested. No effect of Scenario alone was observed (p = 0.505), whereas Parameters had a significant effect (p < 0.001) on the likelihood of physicians fixating on each area of interest. Compared with heart rate, the likelihood of gaze fixation was lower for electrocardiogram (OR = 0.14; 95% CI: 0.04–0.50), respiratory rate (0.16; 0.04–0.59), respiratory curve (0.04; 0.01–0.12), blood pressure (0.07; 0.02–0.24), and temperature (0.04; 0.01–0.15).

At 0–10 s, the interaction between Parameter and Scenario could not be tested. No effect of Scenario alone was observed (p = 0.056), whereas Parameters had a significant effect (p < 0.001) on the likelihood of physicians fixating on each area of interest. Compared with heart rate, the likelihood of gaze fixation was lower only for the respiratory curve (OR = 0.07; 95% CI: 0.02–0.29).

An additional analysis incorporating physician experience (completion vs. non-completion of a fellowship in neonatology or pediatric intensive care) showed no significant interaction between experience and parameters across the study periods (global test: p > 0.05). A significant interaction between scenario and experience was observed in the 0–3 s (p = 0.026) and 0–5 s (p = 0.001) study periods. In the 0–10 s period, experience was identified as an independent predictor of visual fixation, with no significant interaction effects (Supplementary Table S1).

## Discussion

This study evaluated the visual attention of pediatricians when interpreting a multiparameter monitor at 0–3, 0–5, and 0–10 s. Heart rate attracted the attention of most physicians, with 92% of them fixating their gaze on this parameter within 0–3 s, 96% within 0–5 s, and 98% within 0–10 s. After heart rate, parameters related to oxygenation received more visual attention. Compared with heart rate, the likelihood of fixating on the pulse wave did not differ across the three periods, whereas fixation on oxygen saturation was lower only at 0–3 s. Other parameters captured attention only after physicians had looked at heart rate and oxygenation. Overall, when evaluating the monitor display, physicians first focused on heart rate, followed by the pulse wave and oxygen saturation, and subsequently by electrocardiogram, respiratory rate, respiratory curve, blood pressure, and temperature.

Heart rate is a key indicator of cardiorespiratory status and usually requires immediate intervention. For this reason, it initially attracts physicians' attention, regardless of whether it is altered or not. Beyond its clinical relevance, its placement in the upper right corner of the monitor panel may have contributed to an increased visual attention, as human gaze tends to follow a hierarchical pattern in which stimuli are prioritized from top to bottom [9]. The use of green font on a black background may also have enhanced readability, as this color combination is more eye-catching than black text on a green background [10]. In addition, the numerical display of heart rate facilitates rapid recognition and demands less cognitive effort than graphical representations such as curves. Although dynamic stimuli generally attract more visual attention, they require greater cognitive processing, which further supports the attractiveness of heart rate as a parameter for quick interpretation [11].

After heart rate, the parameters most likely to attract visual fixation were those related to oxygenation: the pulse wave and oxygen saturation, regardless of whether oxygen saturation was altered or not. This preference may be explained by their clinical importance in managing neonatal hypoxia and hyperoxia, conditions associated with serious adverse outcomes such as periventricular leukomalacia, peri‑intraventricular hemorrhage, retinopathy of prematurity, and bronchopulmonary dysplasia [12]. As with heart rate, their positioning at the top of the monitor and the use of yellow font may have further enhanced visual attention, since yellow on black provides high readability [13].

Visual fixation on the electrocardiogram occurred only after physicians had observed heart rate and oxygenation parameters, regardless of whether the heart rate was altered or not, reflecting clinical prioritization of vital signs related to clinical urgency and requiring immediate intervention [14]. Besides that, electrocardiographic changes are typically associated with arrhythmias, which are rare in the neonatal period, and their interpretation demands greater cognitive effort due to their dynamic nature. This may have reduced their initial visual appeal to participants [15].

Respiratory rate and the respiratory curve also failed to capture physicians’ initial gaze when evaluating the monitor. This may reflect the fact that, in clinical practice, respiration is frequently assessed directly at the bedside by observing intercostal indrawing and chest retractions. A study monitoring the visual attention of nurses and nursing students in a simulated scenario found that, when assessing newborns’ respiration and heart rate, their gaze was most often directed to the chest and abdomen rather than to the monitor [16]. The lower placement of these parameters on the screen and their display in white font may also have contributed to their reduced visual priority.

The likelihood of physicians directing their gaze to blood pressure increased progressively across the three study periods. This pattern may reflect the greater cognitive complexity involved in interpreting this parameter, which requires analyzing three distinct values (systolic, diastolic, and mean pressure) and recalling neonatal reference ranges that vary by gestational age [17]. Another possible explanation for the reduced initial attention to blood pressure is that, in clinical practice, physicians often rely on nursing staff for updated values in critically ill patients, which may lessen their direct consultation of the monitor. In addition, when measured by the oscillometric method, the monitor displays only the most recent reading, unlike invasive monitoring, which provides continuous data.

Temperature exhibited a pattern of visual attention similar to that of respiratory parameters and blood pressure, even when other parameters changed, such as heart rate, which increases in response to hyperthermia, indicating that this parameter attracts relatively little attention from physicians. This may be explained by the fact that most critically ill neonates remain in incubators equipped with thermal servo-control systems, which minimize large fluctuations in body temperature [18]. Consequently, temperature may be perceived as less urgent, leading to a lower priority during monitor evaluation.

Consistent with the present study, one investigation examined the visual attention of anesthesiologists during a simulation of postpartum hemorrhage. On the multiparameter monitor, heart rate received the greatest number of fixations, followed by blood pressure, oxygen saturation, respiratory rate, electrocardiogram, pulse wave, and respiratory curve [19]. This observation highlights that dynamic elements on the monitor may demand increased cognitive processing, which can influence patterns of visual attention. In contrast, another study evaluating physicians and nurses during positive pressure ventilation in a neonatal resuscitation simulation found that 33% of visual attention was directed toward curves and 20% toward numerical values, likely because interpreting curves requires greater cognitive effort [20]. The discrepant result stems primarily from the neonatal resuscitation simulation scenario, in which a newborn's manikin was being ventilated with positive pressure via a mask, and so, the main objective was to assess the adequacy of pulmonary ventilation. Of the 33% of the time participants focused their gaze on the curves, 70% of their visual attention was directed to the exhaled tidal volume to assess ventilation.

The different monitor scenarios had no impact on physicians' likelihood of fixating on the various vital signs. This finding suggests that physicians display a consistent pattern of vital sign assessment on the monitor panel, regardless of vital sign changes. Studies in cognitive psychology indicate that human visual attention involves hierarchical processing, in which stimuli are prioritized from top to bottom [9]. Thus, physicians show a consistent top-to-bottom gaze pattern when assessing vital signs on the multiparameter monitor, regardless of whether the vital signs are normal or altered.

The role of clinical experience in visual attention appears to be dynamic rather than static. During the 0–3 s interval of monitor evaluation, visual behavior seems to be predominantly stimulus-driven, with no significant differences between levels of experience. In the 0–5 s interval, experience interacts with monitor scenarios, suggesting that they may differentially shape perceptual strategies. By the 0–10 s interval, however, experience emerges as a significant predictor of visual fixation, indicating a shift toward more internally guided and expertise-driven processing. These findings suggest that visual attention in clinical monitoring is not determined solely by expertise, but rather by a temporal interplay among parameters, scenarios, and cognitive processes. This dynamic perspective may help explain inconsistencies in previous studies that have treated visual attention as a static construct and underscores the importance of optimizing human-machine interfaces in the critical care environment.

The visual tracker used in this experiment proved to be a reliable device, capturing eye signals 96–97% of the time across the three analyzed periods (0–3, 0–5, and 0–10 s). Barros et al [21]. employed a 7-second interval to evaluate adults’ visual attention when assessing newborn pain from facial images at rest and during painful procedures. However, using the same database, Orsi et al [22]. found that maximum pupillary dilation, which reflects greater cognitive effort [23] and corresponds to the moment of clinical decision-making, occurred within just two seconds. Given that the monitor panel presents both static stimuli (numerical values) and dynamic stimuli (curves), the analysis in this study began at 3 s, with additional assessments at 5 and 10 s. The 10-second endpoint was chosen to allow sufficient time for a complete evaluation of all parameters displayed on the monitor. The results support this approach, as over 90% of physicians fixated on most parameters within the 10-second period. In the 10-second period, the segmentation of the analyses into the 0–3, 0–5, and 0–10 s periods was random.

To our knowledge, this study is the first to investigate physicians’ visual attention when evaluating a multiparameter monitor panel in a neonatal intensive care setting. However, several limitations should be considered. The research was conducted in a controlled environment, lacking typical NICU conditions such as alarms, background noise, and staff movement, which may limit the generalizability of the findings. The monitor was presented via video on a computer screen, with dimensions and viewing distance differing from those in an actual ICU, as well as variations in gaze angle, since hospital monitors are typically positioned above the patient’s bed. Besides that, the authors included only three clinical situations (normal vital signs, tachycardia, and hyperoxia) and did not consider the overlapping of parameter alterations, which limits the generalization of the results to other situations. Furthermore, only one monitor model (Efficia CM 120®) was used, which may limit the applicability of these results to monitors with different interfaces.

Despite these limitations, the results of this study may inform the training of professionals working in the NICU, emphasizing the need to direct visual attention toward less frequently monitored parameters, such as the electrocardiogram, respiratory variables, blood pressure, and temperature. Adequate training can support faster and more accurate clinical decision-making, ultimately improving patient outcomes [24]. The findings also underscore the potential for optimizing the design of multiparameter monitors, particularly regarding the layout of parameters. User interfaces aligned with cognitive principles can facilitate the interpretation of vital signs, promoting quicker and safer clinical decisions [25].

Further research is needed to validate these findings in bedside studies. Additionally, analyzing gaze trajectories during monitoring and identifying areas of interest associated with peak cognitive demand, such as moments of maximum pupillary dilation, which reflect clinical decision-making [23], could provide valuable insights for optimizing monitoring processes and professional training. Experiments can also be carried out by altering the positioning of the information on the monitor panel, aiming to identify the influence of the position on the visual focus of attention of the physicians. Furthermore, experiments could be conducted to identify whether the physician's experience time in the Neonatal ICU interferes with visual focus when evaluating a multiparameter monitor panel.

In conclusion, in a simulation scenario in which physicians evaluated the panel of a multiparameter monitor presented on a computer screen, the observed progression of visual attention suggests that physicians initially prioritize numerical parameters located at the top of the monitor that require rapid interpretation, such as heart rate and oxygen saturation, which often fluctuate in critically ill newborns. More complex parameters, including the electrocardiogram, respiratory curve, and blood pressure, which demand greater cognitive effort, are generally attended to afterward. This pattern reflects a visual prioritization strategy influenced by clinical importance, panel location, and the complexity of the information, with direct implications for optimizing clinical decision-making.

## Funding

Fundação de Amparo à Pesquisa do Estado de São Paulo (Fapesp) –grant #2012/50157-0 and 2018/13076-9, and Coordenação de Aperfeiçoamento de Pessoal de Nível Superior (CAPES). The funders did not participate in any part of the research.

## Data availability

The data that support the findings of this study are available from the corresponding author.

## Conflicts of interest

The authors declare no conflicts of interest.

## Appendix Supplementary materials

Image, application 1
