# Supplementary material for: What most captures the physician's interest when evaluating a multiparameter monitor in a Neonatal ICU? – A simulation study
Source: J Pediatr (Rio J). 2026 Apr 26;102(4):101548. doi: 10.1016/j.jped.2026.101548 (PMC13112247; doi:10.1016/j.jped.2026.101548)
Supplement: Supplementary file 1 [file mmc1.docx]

**JPED-D-25-00610**

**Supplementary Table S1**

**Supplementary Table S1** Generalized estimating equations (GEE) models including physician experience (doctors with vs. without specialization in neonatology or pediatric intensive care) and interaction with parameters and scenario, according to study periods.

| **Time** | **Terms** | **Variable** | **OR (95% CI)** | **p-value** |
| --- | --- | --- | --- | --- |
| **Time 0 – 3s** | **Interaction terms** | Parameter X Experience (global test) | - | 0.408 |
|  |  | Monitor X Experience (global test) | - | 0.026 |
|  | **Main effects** | Parameter (global test) | - | <0.001 |
|  |  | Monitor (global test) | - | 0.057 |
|  |  | Experience on NICU/PICU (yes vs. no) | 1.96 (0.96 – 3.99) | 0.063 |
|  | **Interaction with monitor** | Monitor 2 | 2.42 (1.13 – 5.19) | 0.024 |
|  |  | Monitor 3 | 0.70 (0.41 – 1.20) | 0.191 |
|  |  | Monitor 2 X Experience on NICU/PICU | 0.31 (0.11 – 0.86) | 0.025 |
|  |  | Monitor 3 X Experience on NICU/PICU | 1.09 (0.41 – 2.87) | 0.862 |
| **Time 0 – 5s** | **Interaction terms** | Parameter X Experience (global test) | - | 0.356 |
|  |  | Monitor X Experience (global test) | - | 0.001 |
|  | **Main effects** | Parameter (global test) | - | <0.001 |
|  |  | Monitor (global test) | - | 0.067 |
|  |  | Experience on NICU/PICU (yes vs. no) | 2.85 (1.25 – 6.49) | 0.013 |
|  | **Interaction with monitor** | Monitor 2 | 2.43 (1.20 – 4.91) | 0.014 |
|  |  | Monitor 3 | 0.56 (0.34 – 0.93) | 0.024 |
|  |  | Monitor 2 X Experience on NICU/PICU | 0.21 (0.07 – 0.65) | 0.007 |
|  |  | Monitor 3 X Experience on NICU/PICU | 1.33 (0.47 – 3.77) | 0.597 |
| **Time 0 – 10s** | **Interaction terms** | Parameter X Experience (global test) | - | not estimable |
|  |  | Monitor X Experience (global test) | - | 0.106 |
|  | **Main effects** | Parameter (global test) | - | <0.001 |
|  |  | Monitor (global test) | - | 0.044 |
|  |  | Experience on NICU/PICU (yes vs. no) | 2.38 (1.26 – 4.49) | 0.008 |

GEE models with binomial family and logit link were used, accounting for repeated measures within individuals (exchangeable correlation structure).

Results are expressed as odds ratios (OR) and 95% confidence intervals (95%CI).

Parameters were compared using heart rate as reference.

Experience was categorized as doctors with vs. without specialization in neonatology (NICU) or pediatric intensive care (PICU)

Global tests correspond to Wald chi-square tests for each factor.

Some interaction terms between parameters and experience could not be estimated due to quasi-complete separation and collinearity, resulting from sparse data and perfect prediction in specific parameter–experience combinations. These terms were omitted to ensure model stability and interpretability.
